# The prognostic role of coagulation markers in the progression and metastasis of laryngeal squamous cell carcinoma

**DOI:** 10.1186/s12885-023-11381-5

**Published:** 2023-09-25

**Authors:** Qiongling Huang, Jing Chen, Yanjun Huang, Yu Xiong, Jiao Zhou, Yizheng Zhang, Ming Lu, Weipeng Hu, Feng Zheng, Chaohui Zheng

**Affiliations:** 1 Department of Otolaryngology, the Second Affiliated Hospital, Fujian Medical University, Quanzhou, 362000 Fujian Province China; 2 Department of Neurosurgery, the Second Affiliated Hospital, Fujian Medical University, Quanzhou, 362000 Fujian Province China; 3 Quanzhou Medical College, Quanzhou, 362000 Fujian Province China

**Keywords:** Laryngeal squamous cell carcinoma, Platelet index, Coagulation index, Fibrinogen, Histopathological features

## Abstract

**Background:**

The application of coagulation-related markers in laryngeal squamous cell carcinoma(LSCC) remains unclear. This study explored the prognostic role of coagulation markers in the progression and metastasis of LSCC.

**Methods:**

Coagulation markers of patients with LSCC receiving surgery in the Second Affiliated Hospital of Fujian Medical University in China, from January 2013 to May 2022 were retrospectively analyzed and compared with those of contemporary patients with benign laryngeal diseases. The relationship between clinicopathological features of LSCC and coagulation markers was analyzed with the chi-square and rank sum tests. The ROC curve analysis was utilized to evaluate the diagnostic efficacy of seven coagulation markers for LSCC and its different clinicopathological features, and to find the optimal cutoff value of each coagulation marker.

**Results:**

303 patients with LSCC and 533 patients with benign laryngeal diseases were included in the present analysis. Compared to the control group, prothrombin time (PT) (p < 0.001), activated partial thromboplastin time (APTT) (p = 0.001), and Fib (p < 0.001) in patients with LSCC were significantly higher, while mean platelet volume (MPV) (p < 0.001) was significantly shorter. Significant increases were detected in PT (Z = 14.342, p = 0.002), Fib (Z = 25.985, p < 0.001), platelet count (PC) (Z = 12.768, p = 0.005), PCT (Z = 9.178, p = 0.027), MPV (F = 2.948, p = 0.033) in T4 stage. Fib had the highest prognostic value among the seven coagulation markers in different T stages (AUC = 0.676, p < 0.001), N stages (AUC = 0.717, p < 0.001), tumor stage (AUC = 0.665, p < 0.001), differentiation degree (AUC = 0.579, p = 0.022), and neurovascular invasion (AUC = 0.651, p = 0.007). Fib (Z = 25.832, p < 0.001), PC (Z = 23.842, p < 0.001), and PCT (Z = 20.15, p < 0.001) in N1 and N3 stages were significantly higher than in N0 stage. PT (Z = 12.174, p = 0.007), Fib (Z = 23.873, p < 0.001), PC (Z = 17.785, p < 0.001), and PCT (Z = 14.693, p = 0.002) were significantly higher in stage IV than in stage I and II. APTT (Z=-1.983, p = 0.047), Fib (Z=-2.68, p = 0.007), PC (Z=-2.723, p = 0.006), and PCT (Z=-2.592, p = 0.01) increased significantly when the tumor invaded neurovascular tissue.

**Conclusions:**

Coagulation markers have the potential to act as biomarkers for predicting pathological features of LSCC. The high level of Fib was helpful for the diagnosis of LSCC and the detection of advanced LSCC.

**Trial registration:**

Not applicable.

**Supplementary Information:**

The online version contains supplementary material available at 10.1186/s12885-023-11381-5.

## Background

Laryngeal cancer is one of the most common malignant tumors in the head and neck. The incidence of laryngeal cancer accounts for 1–5% of all tumor incidence, of which laryngeal squamous cell carcinoma (LSCC) accounts for 95–98% [1–3]. According to the International Agency for Research on Cancer estimates, there were about 177,400 new cases of laryngeal cancer worldwide in 2018; however, the incidence of the disease is expected to rise in the future [4]. Because the primary site of laryngeal cancer is relatively hidden, attention is generally paid when symptoms occur [5]. Although the treatment of laryngeal cancer is constantly improving, many patients are still diagnosed at a late stage with poor prognoses [1, 6]. Therefore, early diagnosis of LSCC is critical.

Recently, coagulation-related parameters have been proven important in tumors’ occurrence, development, and metastasis [7, 8]. Previous studies have reported that hypercoagulability in patients with malignant tumors may promote tumor invasion and metastasis [9, 10]. In addition, unique diagnostic applications and prognostic significance of coagulation markers have been observed in various malignancies, including colorectal, lung, breast, and gastric cancer [8]. Nevertheless, the application of coagulation-related markers in laryngeal cancer remains unclear. In the present study, we sought to explore the potential application of coagulation markers in LSCC and evaluate the association between these markers and tumor pathological features.

## Methods

### Patients and data collection

Between January 2013 and May 2022, consecutive patients who were initially diagnosed with LSCC and underwent surgery in the Second Affiliated Hospital of Fujian Medical University in China were considered for inclusion in the present analysis. All patients with LSCC were staged according to the American Joint Committee on Cancer 8th Edition (UICC/AJCC) TNM staging criteria [11]. The TNM Staging System is based on the extent of the tumor (T), the extent of spread to the lymph nodes (N), and the presence of metastasis (M) [12]. According to this staging system, stages I and II were considered early stages, and stages III and IV were considered advanced stages.

Exclusion criteria were defined as (1) preoperative adjuvant therapy, including radiotherapy, chemotherapy, immunotherapy, and endocrine therapy (2) postoperative pathological diagnosis of non-LSCC (3) combined with infection, liver and kidney disease, blood system disease, and other acute and chronic diseases affecting coagulation and platelet index (4) oral drugs affecting coagulation and platelet index (5) preoperative distant metastasis (6) combined with other non-LSCC tumor history (7) incomplete clinical data (8) giving up treatment. Laryngeal disease patients with a pathologically benign diagnosis were included as the control group. All clinical data were obtained from the medical record system, including patient age, gender, laboratory parameters and histopathological features.

Patients in the LSCC group underwent biopsy via laryngoscopy pre-operatively to determine the pathological type and the cT stage. The cT stage is divided into T1, T2, T3 and T4 according to the extent of tumor invasion. The cN stage was determined with CT and MRI examinations, which were divided into N0, N1, N2, and N3 according to cervical lymph node metastasis. All patients underwent detailed examinations before surgery to exclude distant metastasis and systemic diseases. A team of experienced pathologists standardized histopathological examination of all postoperative tumor tissue specimens according to the UICC classification [11].

### Ethics

This study is based on the current version of the Helsinki Declaration and good clinical practice guidelines. This retrospective study was approved by the Medical ethics committee of the Second Affiliated Hospital of Fujian Medical University (No. 301, 2022) and exempted from informed consent.

### Inspection instruments and detection methods 

The next morning after admission, fasting venous blood was taken from the subjects, and prothrombin time (PT), activated partial thromboplastin time (APTT), fibrinogen (Fib), platelet count (PC), plateletcrit (PCT), mean platelet volume (MPV) and platelet distribution width (PDW) were detected after heparin anticoagulation. The coagulation index was detected by STA-R MAX (Stago, France) automatic coagulation analyzer, and the blood routine was detected by the XN-1000 (Hessenmeikang, Japan) hematology analyzer. The anticoagulant was a 109 mmol/L sodium citrate solution. In the morning, 2 ml of fasting venous blood of all patients was collected using a special negative-pressure vacuum tube for coagulation. After anticoagulation with 1 : 9 sodium citrate, the coagulation function test was performed according to the operation specifications, and PT, APTT, and Fib were detected and recorded. Testing work was completed within 2 h after blood collection in accordance with the instrument operating procedures and reagent requirements for testing.

### Statistical analysis

The distribution pattern of the variables was analyzed with the test of Kolmogorov-Smirnov. All quantitative data conforming to normal distribution were expressed as mean ± standard deviation (${\bar x}$± s), and group design data were calculated by independent sample t-test. The measurement data with non-normal distribution were described by median M (1/4, 3/4), and the non-parametric rank sum test (Mann-Whitney U) was used for inter-group comparison and post-hoc pairwise comparison. Qualitative data were described by frequency and calculated by the chi-square test and Fisher’s exact test. Logistic regression was used for multivariate analysis of meaningful indicators in univariate analysis. Spearman Rank correlation analysis was used to evaluate the correlation between the test indicators. the receiver operating curve (ROC) was utilized to evaluate the diagnostic efficacy of seven coagulation markers for LSCC and its different clinicopathological features, and to find the optimal cutoff value of each coagulation marker. All data were analyzed by SPSS 26.0 statistical software (IBM, Armonk, NY, USA), and p < 0.05 indicated that the difference was statistically significant.

## Results

### Clinical and histopathological features of the patients

**Fig. 1:**
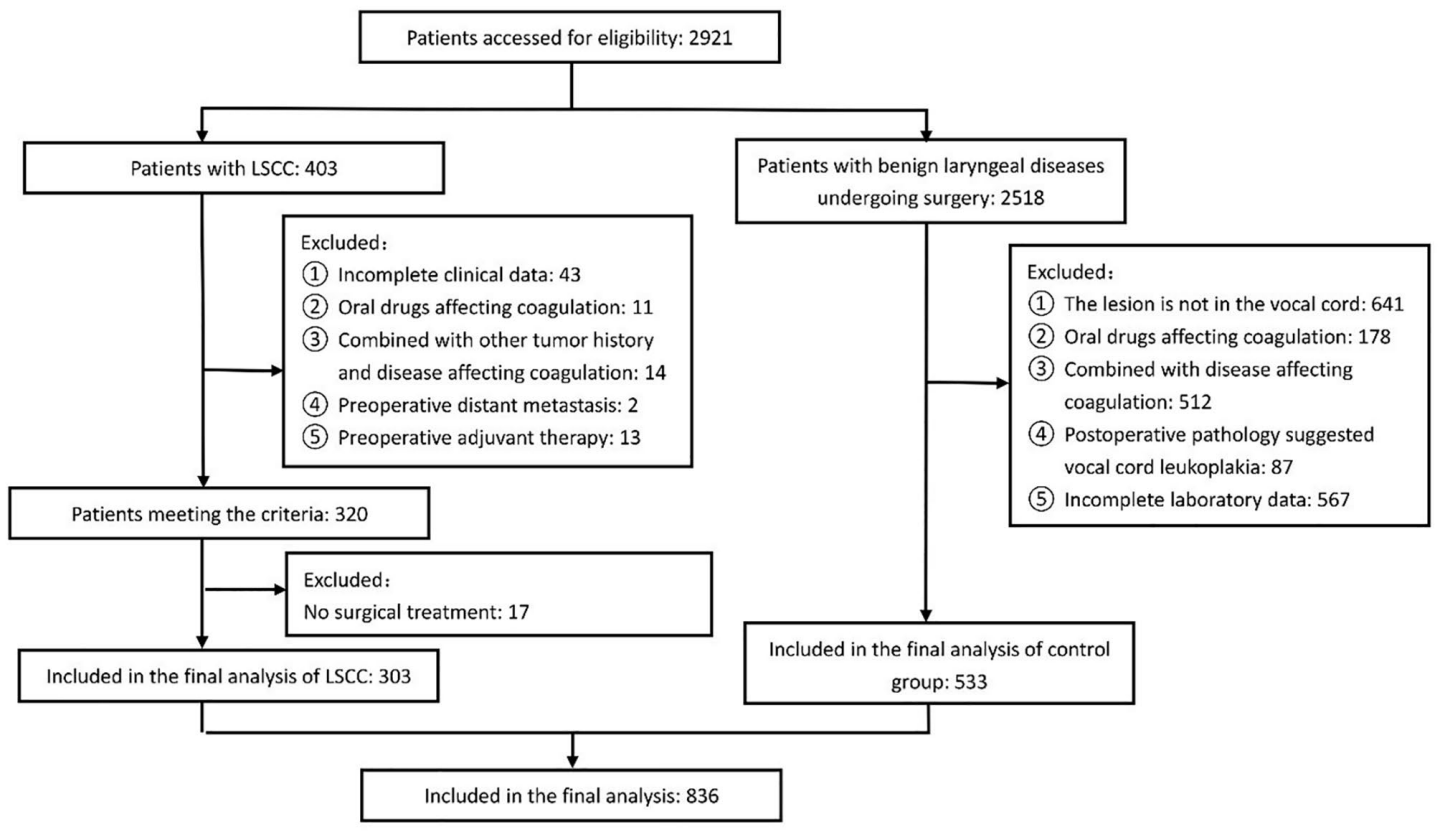
Flow chart of patient inclusion and exclusion. LSCC: laryngeal squamous cell carcinoma

Between January 2013 and May 2022, a total of 2,921 cases, including 403 patients with LSCC and 2,518 patients with benign laryngeal disease, underwent excision surgery of laryngeal lesions in our institute. In the LSCC group, patients not having complete clinical data (n = 43), combined with other tumor history and disease affecting coagulation (n = 14), receiving preoperative adjuvant therapy (n = 13), having preoperative distant metastasis (n = 2), refusing surgical treatment (n = 17), and taking oral drugs affecting coagulation (n = 11) were excluded. Among the patients with benign laryngeal diseases who underwent excision surgery of laryngeal lesions, patients not having complete clinical data (n = 567), combined with other diseases affecting coagulation (n = 512), lesions not located in the vocal cords (n = 641), taking oral anticoagulants (n = 178), and suggesting laryngeal leukoplakia in postoperative pathology (n = 87) were excluded. Finally, a total of 303 LSCC patients and 533 patients with benign laryngeal diseases were eligible and included in the present analysis, as shown in Fig. 1. The clinical characteristics and coagulation parameters of patients are detailed in Table 1.

**Table 1:** Clinical features of laryngeal carcinoma group and control group

Characteristic			Patients with LSCC(n = 303)	Control Group(n = 533)	χ2/Z	p-value
**Gender**					263.033	< 0.001*
Female			10(3.3%)	330(61.9%)		
Male			293(96.7%)	203(38.1%)		
**Age(years)**					275.048	< 0.001*
< 60			116(38.3%)	484(90.8%)		
≥ 60			187(61.7%)	49(9.2%)		
**Smoking**					20.23	< 0.001*
No			49(16.2%)	161(30.2%)		
Yes			254(83.8%)	372(69.8%)		
**Tumor localization**					-	-
Supraglottic			52(17.2%)	-		
Glottic			244(80.5%)	-		
Subglottic			7(2.3%)	-		
**Pathologic T stage**					-	-
T1			132(43.6%)	-		
T2			76(25.1%)	-		
T3			51(16.8%)	-		
T4			44(14.5%)	-		
**Pathologic N stage**					-	-
N0			249(82.2%)	-		
N1			11(3.6%)	-		
N2			14(4.6%)	-		
N3			29(9.6%)	-		
**Tumor stage**					-	-
I			130(42.9%)	-		
II			69(22.8%)	-		
III			34(11.2%)	-		
IV			70(23.1%)	-		
**Histological grade**					-	-
Poorly			15(5.0%)	-		
Moderately			179(59.1%)	-		
Well			109(35.9%)	-		
**LVI or/and PNI**					-	-
No			274(90.4%)	-		
Yes			29(9.6%)	-		
**Coagulation markers**						
**PT(S)**			12(11.4,12.7)	11.714 ± 0.93	-4.837	< 0.001*
**APTT(S)**			29.3(26.8,31.7)	28.372 ± 3.742	-3.254	0.001*
**Fib(g/L)**			3.42(2.84,4.22)	2.67(2.25,3.07)	-12.572	< 0.001*
**PC(10** ^9^ **/L)**			242(203,287)	242(206,290)	-0.031	0.975
**PCT(%)**			0.24(0.2,0.29)	0.25(0.22,0.29)	-1.702	0.089
**MPV(fL)**			9.952 ± 0.917	10.2(9.6,10.9)	-4.189	< 0.001*
**PDW(fL)**			11.8(10.8,15.6)	12.4(11.0,15.4)	-1.609	0.108

PT: prothrombin time, APTT: activated partial thromboplastin time, Fib: fibrinogen, PC: platelet count, PCT: plateletcrit, MPV: mean platelet volume, PDW: platelet distribution width, LSCC: laryngeal squamous cell carcinoma, LVI: lymphovascular invasions, PNI, perineural invasions

* indicates that the difference is statistically significant. - represents not available

### Comparison of blood indexes between LSCC group and control group

**Fig. 2:**
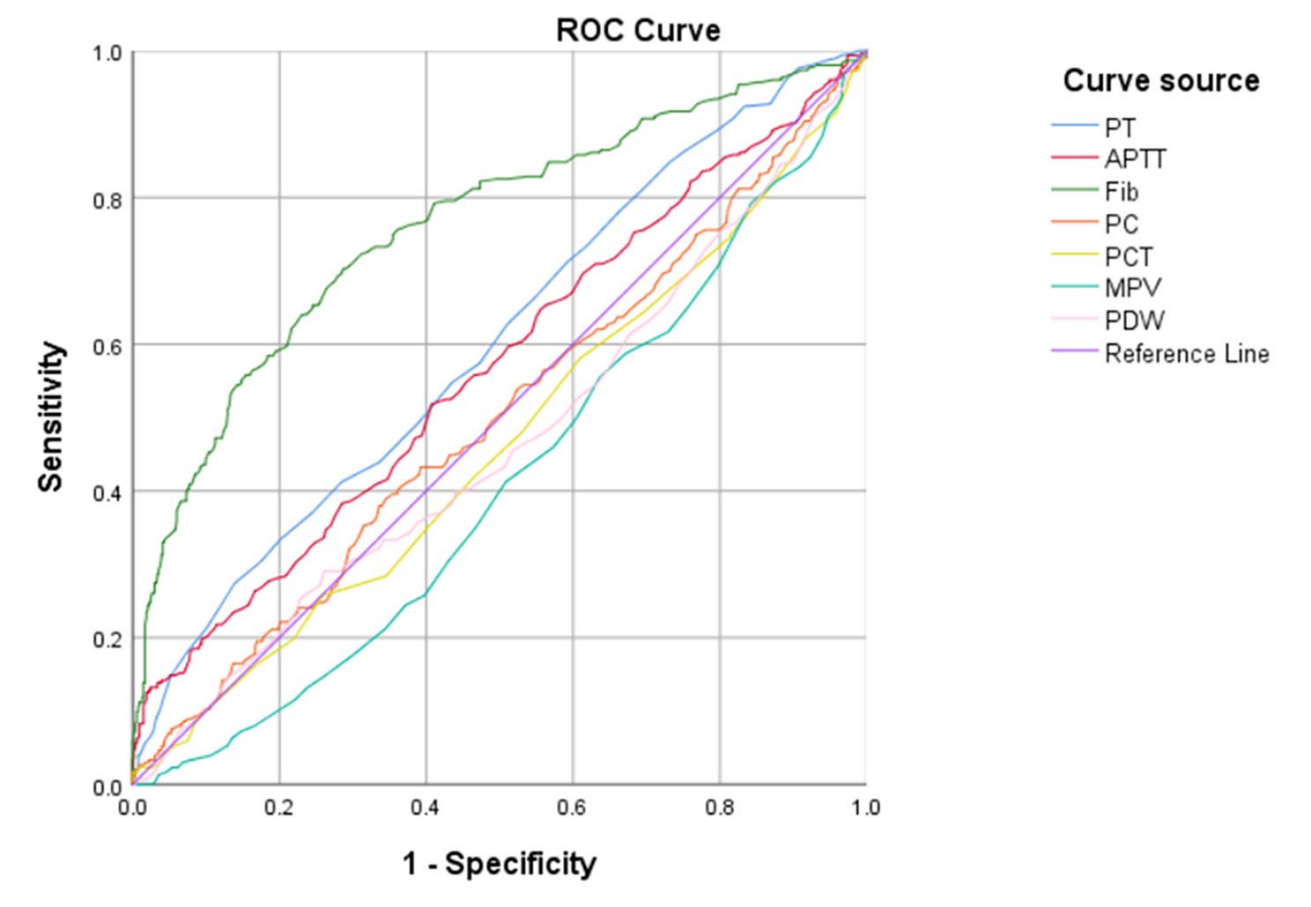
ROC curve of seven coagulation markers in predicting laryngeal squamous cell carcinoma. PT: AUC was 0.6, the optimal cut-off value was 12.65, the sensitivity was 27.4%, and the specificity was 86.1%. APTT: AUC was 0.568, the optimal cut-off value was 29.15, the sensitivity was 51.8%, and the specificity was 59.3%. Fib: AUC was 0.761, the optimal cut-off value was 2.975, the sensitivity was 70.3%, and the specificity was 41.6%. MPV: AUC was 0.413, the optimal cut-off value was 8.15, the sensitivity was 98.7%, and the specificity was 1.5%. ROC: receiver operating curve, AUC: area under the curve, PT: prothrombin time, APTT: activated partial thromboplastin time, Fib: fibrinogen, PC: platelet count, PCT: platelet hematocrit, MPV: mean platelet volume, PDW: platelet distribution width

PT (Z=-4.837, p < 0.001), APTT (Z=-3.254, p = 0.001), Fib (Z=-12.572, p < 0.001), and MPV (Z=-4.189, p < 0.001) in the LSCC group were significantly higher than in the control group. No significant difference was detected in PC (Z=-0.031, p = 0.975), PCT (Z=-1.702, p = 0.089), and PDW (Z=-1.609, p = 0.108) between the two groups. PT was positively correlated with APTT (r = 0.505, p < 0.001) and Fib (r = 0.223, p < 0.001). APTT was positively correlated with Fib (r = 0.915, p < 0.001), while MPV was negatively correlated with PT (r=-0.146, p < 0.001), APTT (r=-0.083, p = 0.017), and Fib (r=-0.257, p < 0.001). Four coagulation markers with statistically significant differences in univariate analysis were included in the logistic regression model. After adjusting for PT, APTT and MPV, Fib was significantly associated with the risk of LSCC in the logistic regression model (OR = 3.178, 95%CI:2.545–3.968, p < 0.001). (Supplement Table 3). ROC analysis showed that PT (p < 0.001, 95%CI: 0.561–0.64), APTT (p = 0.001, 95%CI: 0.527–0.609), Fib (p < 0.001,95%CI: 0.726–0.796), and MPV (p < 0.001, 95%CI: 0.374–0.425) presented statistically significant differences between the LSCC group and the control group. The differences of coagulation markers in different subregions of LSCC were further compared, and the results showed that PC (Z = 12.493, p = 0.002) and MPV (F = 4.985, p = 0.007) presented significant statistical differences in the three types (supraglottic/glottic/subglottic type). PC of the glottic type was significantly lower than that of the supraglottic type (p < 0.05), while MPV was significantly higher than that of the other two types (p < 0.05). The Area Under the ROC (AUC) and cut-off values of the coagulation parameters in the two groups are shown in Fig. 2 and Supplementary Table 1.

### Potential predictive performance of coagulation markers for postoperative pathology

By comparing the coagulation indexes of patients with different degrees of differentiation of LSCC, except Fib (Z = 7.721, p = 0.021), no significant differences were detected in the other six indicators. In different T stages of LSCC, there were significant differences in PT (Z = 14.342, p = 0.002), Fib (Z = 25.985, p < 0.001), PC (Z = 12.768, p = 0.005), PCT (Z = 9.178, p = 0.027), and MPV (F = 2.948, p = 0.033). Similarly, significant differences were detected in Fib (Z = 25.832, p < 0.001), PC (Z = 23.842, p < 0.001), and PCT (Z = 20.15, p < 0.001) in different N stages, among which N1 stage and N3 stage were significantly higher than N0 stage (p < 0.05). In different tumor stages, significant differences were observed in PT (Z = 12.174, p = 0.007), Fib (Z = 23.873, p < 0.001), PC (Z = 17.785, p < 0.001), and PCT (Z = 14.693, p = 0.002). Based on whether neurovascular invasion occurs, further subgroup analysis showed that APTT (Z=-1.983, p = 0.047), Fib (Z=-2.68, p = 0.007), PC (Z=-2.723, p = 0.006), and PCT (Z=-2.592, p = 0.01) were significantly increased in the presence of neurovascular invasion. Subgroup analysis of coagulation markers in histopathological features as shown in Table 2.

**Table 2:** Subgroup analysis of coagulation markers in histopathological features

Variables	PT(s)	APTT(s)	Fib(g/L)	PC(10^9^/L)	PCT(%)	MPV(fL)	PDW(fL)
**Pathologic T stage**							
T1	12(11.4,12.55)	28.8(26.75,31.1)	3.26(2.73,3.77)	239.5(205,279.5)	0.24(0.21,0.28)	9.95 ± 0.83	12.25(10.85,15.8)
T2	11.8(11.2,12.35)	28.7(25.7,32.7)	3.42(2.83,3.98)	230.5(186.5,276)	0.24(0.2,0.27)	10.18 ± 1.04	12(10.9,14.5)
T3	12.2(11.6,13.1)	30.4(27.45,33.1)	3.9(3.11,4.72)^a^	262(208.5,304)	0.24(0.22,0.29)	9.77 ± 0.9^b^	11.7(10.4,13.45)
T4	12.6(11.65,13.15)^b^	29.45(26.95,31.6)	4.27(3.185,5.05)^ab^	278.5(213,322.5)^b^	0.27(0.21,0.32)^b^	9.77 ± 0.91^b^	11.25(10.6,15.75)
Z/F	14.342	2.822	25.985	12.768	9.178	F = 2.948	3.225
p-value	0.002*	0.42	< 0.001*	0.005*	0.027*	0.033*	0.358
**Pathologic N stage**							
N0	12(11.4,12.6)	28.8(26.3,31.6)	3.34(2.78,4)	235(199,277)	0.24(0.2,0.27)	9.99 ± 0.88	11.9(10.8,15.3)
N1	12.8(11.15,13.05)	31.2(27.85,31.7)	4.83(3.93,5.3)^a^	308(281.5,349)^a^	0.29(0.27,0.32)^a^	9.62 ± 1.244	15.4(10.3,15.85)
N2	12.05(11.6,13.1)	30(28.6,31.5)	4.13(3.59,4.85)	283(230,323)	0.28(0.22,0.33)	9.63 ± 1.13	12(9.8,16.1)
N3	12.1(11.7,13.1)	30.4(29.2,33.4)	4.22(3.09,4.69)^a^	279(238,314)^a^	0.27(0.24,0.31)^a^	9.87 ± 1.0	11.6(10.6,15.5)
Z/F	6.76	6.649	25.832	23.842	20.15	F = 1.318	0.74
p-value	0.08	0.084	< 0.001*	< 0.001*	< 0.001*	0.269	0.864
**Tumor stage**							
I	12(11.4,12.5)	28.8(26.7,31.1)	3.29(2.78,3.78)	239.5(205,276)	0.24(0.21,0.27)	9.96 ± 0.83	12.3(10.9,15.8)
II	11.8(11.2,12.4)	28.5(25.5,31.7)	3.41(2.82,3.96)	222(185,275)	0.23(0.2,0.26)	10.11 ± 1.0	11.9(10.8,13.9)
III	12.2(11.6,13.1)	30.35(25.8,33.4)	3.86(2.87,4.69)	231.5(200,301)	0.24(0.2,0.29)	9.91 ± 0.93	11.85(11,15.4)
IV	12.35(11.6,13.1)^b^	30.1(27.8,32.2)	4.11(3.12,4.89)^ab^	276.5(228,318)^ab^	0.27(0.23,0.32)^ab^	9.8 ± 0.97	11.35(10.3,15.7)
Z/F	12.174	4.758	23.873	17.785	14.693	F = 1.341	4.062
p-value	0.007*	0.19	< 0.001*	< 0.001*	0.002*	0.261	0.255
**Histological grade**							
Poorly	12(11.4,12.8)	29.7(27.65,32.85)	4.21(3.51,4.64)	240(205.5,273)	0.25(0.19,0.33)	10.15 ± 1.16	11.7(10.8,14.5)
Moderately	12(11.4,12.8)	29.4(26.8,31.85)	3.51(2.92,4.25)	240(200,284.5)	0.24(0.2,0.29)	9.91 ± 0.92	12.1(10.8,15.55)
Well	12.1(11.4,12.6)	28.8(26.5,31.5)	3.17(2.78,4.02)^a^	245(206,294)	0.24(0.21,0.28)	9.99 ± 0.88	11.6(10.6,15.6)
Z/F	0.118	1.312	7.721	0.216	0.198	0.685	0.319
p-value	0.943	0.519	0.021*	0.898	0.906	0.505	0.853
**LVI or/and PNI**							
No	12(11.4,12.7)	29(26.6,31.6)	3.41(2.83,4.08)	240(200,281)	0.24(0.2,0.28)	9.96 ± 0.91	11.9(10.8,15.6)
Yes	12.1(11.7,13.1)	30.4(29.2,33.4)	4.22(3.09,4.69)	276(238,314)	0.27(0.24,0.31)	9.88 ± 1.003	11.6(10.6,15.5)
Z/t	-1.5	-1.983	-2.68	-2.723	-2.592	t = 0.490	-0.827
p-value	0.134	0.047*	0.007*	0.006*	0.01*	0.625	0.408

PT: prothrombin time, APTT: activated partial thromboplastin time, Fib: fibrinogen, PC: platelet count, PCT: platelet hematocrit, MPV: mean platelet volume, PDW: platelet distribution width, LVI, lymphovascular invasions, PNI, perineural invasions

* indicates that the difference is statistically significant

^a^ represents comparison with the first term p < 0.05

^b^ represents comparison with the second term p < 0.05

The t value is obtained by independent sample t-test. The Z value is derived from the non-parametric rank sum test. The F value is derived from one-way ANOVA.

Subgroup analysis based on histopathological features showed that Fib had the highest prognostic value among the seven coagulation markers in different T stages (AUC = 0.676, p < 0.001, 95%CI: 0.628–0.807), N stages (AUC = 0.717, p < 0.001, 95%CI : 0.628–0.807), tumor stage (AUC = 0.665, p < 0.001, 95%CI : 0.597–0.734), differentiation degree (AUC = 0.579, p = 0.022, 95%CI : 0.513–0.646), and neurovascular invasion (AUC = 0.651, p = 0.007, 95%CI : 0.542–0.761). Fib (AUC = 0.717, p < 0.001, 95%CI : 0.628–0.807), PC (AUC = 0.724, p < 0.001, 95%CI : 0.636–0.812), and PCT (AUC = 0.702, p < 0.001, 95%CI : 0.609–0.796) are good predictors of lymph node metastasis of LSCC. Fib, PC, and PCT cut-off values were 4.54, 275.5, and 0.351, respectively. Potential predictive performance of coagulation markers for postoperative pathology as shown in Supplementary Table 2.

## Discussion

According to different tumor locations, LSCC was divided into supraglottic, glottic, and subglottic types, of which the most common type was glottic [5]. Different locations of the tumor can affect the clinical manifestations and diagnosis. Therefore, in the cases with tumor-compressed symptoms, including hoarseness, dysphagia, and dyspnea, further examination is required to identify the diagnosis of LSCC [13–15]. Despite the development of diagnosis and treatment technology, the survival rate of LSCC has not improved significantly in the past two decades [7, 16]. LSCC is mostly diagnosed at an advanced stage, which affects life quality postoperatively [17]. Effective prognostic markers are warranted for early diagnosis and treatment of LSCC, which may improve the prognosis.

It has been reported that tumor-induced coagulation dysfunction is related to tumorigenesis and tumor development [18–20]. In the present study, platelet index as a coagulation marker helps diagnose LSCC and has good predictive value for histopathological features. It has been reported that hypercoagulability was positively correlated with tumor invasiveness in patients with malignant tumors [8, 12].

Recent studies have found that coagulation cascades play an important role in the tumor immune microenvironment [21, 22]. Coagulants can interact with the tumor microenvironment and affect tumor progression and tumor immune response [23]. The platelet index is related to platelet morphology and proliferation kinetics [24]. Under inflammatory conditions, platelets significantly increased with the stimulation of inflammatory mediators, including vascular endothelial growth factor-A (VEGF-A), reactive oxygen species (ROS), and IL-6 [25, 26]. Activated platelets promote the release of inflammatory mediators in the tumor microenvironment, recruiting more platelets and inflammatory cells, which leads to local and systemic immunosuppression regarding cancer. Fib can bind to VEGF-A and promote the adhesion of VEGF-A to the surface of tumor cells, contributing to tumor proliferation and angiogenesis [27–29]. Zhang et al. have shown that PC and MPV are of great value in predicting lymph node involvement in esophageal cancer [30]. Our study also found that the PC level in LSCC patients with lymph node metastasis was significantly higher than that in patients without lymph node metastasis, and PC in advanced LSCC was significantly higher than in the early stage.

MPV and PDW are the commonly used indicators for judging platelet volume, and it is recommended to apply them together to diagnose the disease [25]. Inagaki et al. found that patients with non-small cell lung cancer had a lower MPV than those with chronic obstructive pulmonary disease [31]. Our study also confirmed that the MPV of LSCC was significantly lower than that in the control group, and the changes of MPV in different T stages were statistically different, showing a higher level in the T2 stage. This may be because of increased platelet release leading to a decrease in MPV under the stimulation of inflammation.

APTT and PT reflect the content and activity of coagulation factors in internal and external coagulation pathways, respectively [32]. In our study, the PT and APTT of the LSCC group were significantly longer than those of the control group. This is consistent with previous reports, which may be because of body failure caused by tumor depletion affecting the synthesis of coagulation factors [33–35]. In addition, significant differences were detected in PT in different T and TNM stages, with significant prolongation in T4 and stage IV. Hypercoagulability leads to a reduced blood supply and subsequent hypoxia, which is a key factor in tumor growth and metastasis [36].

**Fig. 3:**
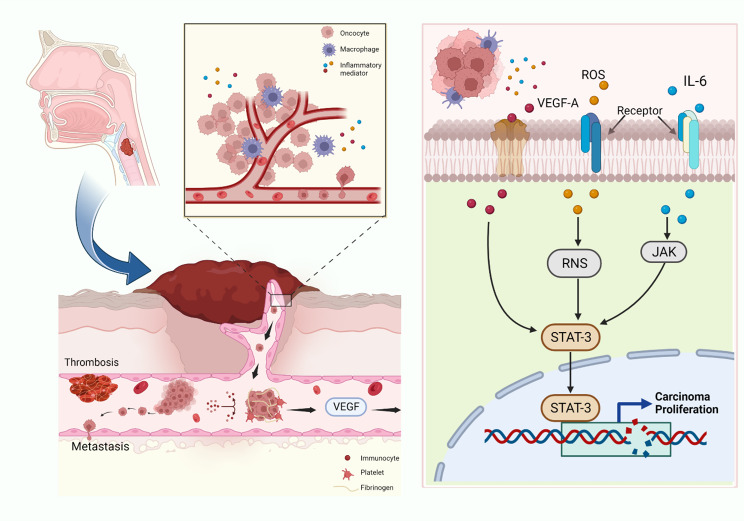
Mechanism of coagulation index in LSCC. Tumor cells invade the surrounding tissue of the primary tumor cells, enter the blood system and are transported to other organs to form metastases. The interaction between tumor cells and platelets allows them to migrate from the primary site to form metastasis, and platelets accumulate around cancer cells to protect them and form immune escape. Fib also promotes platelet aggregation and adhesion around tumor cells to protect them from immune cells. VEGF-A is secreted by tumor cells, indirectly promotes coagulation and changes the coagulation properties of endothelial cells. VEGF-A binds to Fib, promotes angiogenesis in cancer tissues and leads to cancer growth and metastasis. Platelets significantly increased with the stimulation of VEGF-A, ROS and IL-6. The combination of ROS and RNS and the combination of IL-6 and JAK induce DNA damage leading to genetic instability through the STAT-3 pathway, promote cancer cell proliferation and angiogenesis. VEGF-A: vascular endothelial growth factor-A, Fib: fibrinogen, RNS: reactive nitrogen species, ROS: reactive oxygen species, LSCC: laryngeal squamous cell carcinoma, IL-6: interleukin-6, JAK: janus kinase, STAT-3: signal transducer and activator of transcription-3

Fib is a proinflammatory protein and an important substance in coagulation and thrombosis. The level of Fib reflects the hypercoagulable state of blood, which indicates hematogenous and lymph node metastasis or deeper tumor invasion [37–39]. The combination of ROS, reactive nitrogen species (RNS), IL-6, and JAK induces DNA damage leading to genetic instability through the STAT-3 pathway, promoting cancer cell proliferation and angiogenesis [40–42]. The mechanism of the coagulation index in LSCC is shown in Fig. 3. We found that the level of Fib in LSCC patients with lymph node metastasis was significantly higher than that in LSCC patients without lymph node metastasis. Fib was high in patients with advanced and poorly differentiated LSCC. Accordingly, it was reported that Fib is elevated in various malignancies, including colorectal, lung, breast, and gastric [32, 43, 44]. Moreover, we found that Fib increased more significantly in LSCC patients with poor differentiation, lymph node metastasis, neurovascular invasion, and advanced LSCC.

Compared to patients with early LSCC (stage I/ II), PT, Fib, PC, and PCT in patients with advanced LSCC (stage III/ IV) were different, and Fib, PC, and PCT in stage IV were significantly higher than in stage I and II. APTT, Fib, PC, and PCT levels in LSCC patients with neurovascular invasion were significantly higher than those without neurovascular invasion. Significant differences regarding Fib were detected in different degrees of differentiation, T stage, N stage, tumor stage, and neurovascular invasion. In our logistic regression model, Fib is the most effective coagulation marker for the prognosis of LSCC among the seven coagulation markers. Furthermore, Fib has a better prognosis value for tumor staging, differentiation, lymph node metastasis, and neurovascular invasion among the seven coagulation indicators.

It has been reported that Fib can reduce the sensitivity of tumor cells to chemotherapy, protect tumor cells, and thus promote their proliferation and metastasis [12, 45]. Despite recent reports on the application of anticoagulant therapy using heparin or low molecular weight heparin in LSCC patients with high Fib, its efficacy for LSCC has not been established [46–48]. Therefore, in LSCC patients with high Fib, anticoagulant therapy may also be considered, with the re-evaluation of the chemotherapy sensitivity. The efficacy of anticoagulant therapy for LSCC patients with high Fib levels remains to be further explored.

There are several limitations in the present study. First, our study was conducted retrospectively; thus, any conclusions drawn are subject to the limitations of the respective study design, including recall and observer bias. Secondly, due to the exclusion of patients with infection and other acute and chronic diseases affecting coagulation and platelet index, the assessment of interaction terms in multivariate models, including variables affecting inflammation, was not performed in the present study. Future studies should be focused on this issue. In addition, subgroup analysis based on different primary sites of LSCC could not be achieved in the present study because of the absence of relevant data. Further studies should focus on this issue.

## Conclusions

Hypercoagulability in patients with LSCC is associated with differentiation, lymph node metastasis, and tumor stage. The levels of PC, PCT, MPV, PDW, PT, APTT, and Fib can assist in diagnosing LSCC and detecting advanced-stage disease. Fib significantly increases with the neurovascular invasion of tumor and lymph node metastasis and may be used as a predictor of LSCC for early tumor screening and prognosis assessment. Future studies with larger sample sizes are warranted to confirm this finding.

### Electronic supplementary material

Below is the link to the electronic supplementary material.

Supplementary Material 1

## Data Availability

Data are available from the corresponding authors on reasonable request.

## List of abbreviations

APTT: activated partial thromboplastin time
AUC: area under the receiver operating curve
Fib: fibrinogen
LSCC: laryngeal squamous cell carcinoma
M: metastasis
MPV: mean platelet volume
N: lymph node metastasis
PC: platelet count
PCT: plateletcrit
PDW: platelet distribution width
PT: prothrombin time
RNS: reactive nitrogen species
ROC: receiver operating curve
ROS: reactive oxygen species
T: primary solvent tumor
VEGF-A: vascular endothelial growth factor-A
