# Supplementary material for: The prognostic role of coagulation markers in the progression and metastasis of laryngeal squamous cell carcinoma
Source: BMC Cancer. 2023 Sep 25;23:901. doi: 10.1186/s12885-023-11381-5 (PMC10519099; doi:10.1186/s12885-023-11381-5)
Supplement: Supplementary file 1 — Supplementary Material 1 [file 12885_2023_11381_MOESM1_ESM.docx]

Supplementary Table 1. Predictive value of coagulation markers in laryngeal squamous cell carcinoma

| **Parameters** | **AUC** | **p-value** | **95%CI** | | **Youden Index** | **cut-off** | **Sensitivity** | **Specificity** |
| --- | --- | --- | --- | --- | --- | --- | --- | --- |
| **PT(S)** | 0.6 | <0.001* | 0.561-0.64 | 0.135 | | 12.65 | 0.274 | 0.861 |
| **APTT(S)** | 0.568 | 0.001* | 0.527-0.609 | | 0.111 | 29.15 | 0.518 | 0.593 |
| **Fib(g/L)** | 0.761 | <0.001* | 0.726-0.796 | | 0.416 | 2.975 | 0.703 | 0.416 |
| **PC(10⁹/L)** | 0.499 | 0.975 | 0.458-0.541 | | 0.044 | 266.5 | 0.389 | 0.655 |
| **PCT(%)** | 0.465 | 0.089 | 0.424-0.506 | | 0.017 | 0.47 | 0.017 | 1 |
| **MPV(fL)** | 0.413 | <0.001* | 0.374-0.452 | | 0.002 | 8.15 | 0.987 | 0.015 |
| **PDW(fL)** | 0.467 | 0.108 | 0.425-0.508 | | 0.029 | 15.15 | 0.29 | 0.739 |

AUC: area under the curve, CI: confidence interval, PT: prothrombin time, APTT: activated partial thromboplastin time, Fib: fibrinogen, PC: platelet count, PCT: plateletcrit, MPV: mean platelet volume, PDW: platelet distribution width.

* indicates that the difference is statistically significant.

Supplementary Table 2. Potential predictive performance of coagulation markers for postoperative pathology

| **Variables** | **AUC** | **p-value** | **95%CI** | **Youden Index** | **cut-off** | **Sensitivity** | **Specificity** |
| --- | --- | --- | --- | --- | --- | --- | --- |
| **pT stage** |  |  |  |  |  |  |  |
| PT | 0.625 | <0.001 | 0.555-0.695 | 0.25 | 12.45 | 0.505 | 0.745 |
| APTT | 0.556 | 0.121 | 0.485-0.626 | - | - | - | - |
| Fib | 0.676 | <0.001 | 0.606-0.746 | 0.315 | 3.71 | 0.632 | 0.683 |
| PC | 0.603 | 0.004 | 0.531-0.676 | 0.214 | 257 | 0.579 | 0.635 |
| PCT | 0.595 | 0.008 | 0.523-0.667 | 0.208 | 0.285 | 0.4 | 0.808 |
| MPV | 0.412 | 0.014 | 0.343-0.481 | 0.005 | 7.3 | 1 | 0.005 |
|  |  |  |  |  |  |  |  |
| PDW | 0.442 | 0.104 | 0.371-0.512 | - | - | - | - |
| **pN stage** |  |  |  |  |  |  |  |
| PT | 0.603 | 0.034 | 0.506-0.7 | 0.219 | 12.65 | 0.463 | 0.756 |
| APTT | 0.603 | 0.035 | 0.521-0.684 | 0.24 | 29 | 0.732 | 0.508 |
| Fib | 0.717 | <0.001 | 0.628-0.807 | 0.381 | 4.54 | 0.488 | 0.893 |
| PC | 0.724 | <0.001 | 0.636-0.812 | 0.42 | 275.5 | 0.683 | 0.737 |
| PCT | 0.702 | <0.001 | 0.609-0.796 | 0.351 | 0.351 | 0.561 | 0.79 |
| MPV | 0.364 | 0.005 | 0.263-0.465 | 0.067 | 11.65 | 0.098 | 0.969 |
| PDW | 0.473 | 0.576 | 0.362-0.584 | - | - | - | - |
| **Tumor stage** | |  |  |  |  |  |  |
| PT | 0.615 | 0.001 | 0.546-0.684 | 1 | 16.7 | 0 | 0 |
| APTT | 0.574 | 0.035 | 0.505-0.642 | 0.176 | 29.75 | 0.558 | 0.618 |
| Fib | 0.665 | <0.001 | 0.597-0.734 | 1 | 0.08 | 1 | 0 |
| PC | 0.61 | 0.002 | 0.541-0.679 | 0.209 | 272.5 | 0.49 | 0.719 |
| PCT | 0.613 | 0.001 | 0.544-0.682 | 0.223 | 0.285 | 0.404 | 0.819 |
| MPV | 0.435 | 0.065 | 0.366-0.505 | - | - | - | - |
| PDW | 0.458 | 0.225 | 0.388-0.527 | - | - | - | - |
| **LVI/PNI** |  |  |  |  |  |  |  |
| PT | 0.585 | 0.134 | 0.471-0.698 | - | - | - | - |
| APTT | 0.612 | 0.047 | 0.507-0.717 | 0.266 | 29.15 | 0.759 | 0.057 |
| Fib | 0.651 | 0.007 | 0.542-0.761 | 0.291 | 4.215 | 0.517 | 0.774 |
| PC | 0.654 | 0.006 | 0.551-0.757 | 0.268 | 261.5 | 0.655 | 0.613 |
| PCT | 0.646 | 0.01 | 0.54-0.752 | 0.22 | 0.275 | 0.483 | 0.737 |
| MPV | 0.47 | 0.597 | 0.35-0.59 | - | - | - | - |
| PDW | 0.453 | 0.408 | 0.341-0.566 | - | - | - | - |
| **Histological grade** | |  |  |  |  |  |  |
| PT | 0.511 | 0.76 | 0.444-0.577 | - | - | - | - |
| APTT | 0.534 | 0.323 | 0.468-0.601 | - | - | - | - |
| Fib | 0.579 | 0.022 | 0.513-0.646 | 0.006 | 1.77 | 0.991 | 0.015 |
| PC | 0.489 | 0.753 | 0.422-0.557 | - | - | - | - |
| PCT | 0.499 | 0.983 | 0.432-0.567 | - | - | - | - |
| MPV | 0.485 | 0.675 | 0.419-0.552 | - | - | - | - |
| PDW | 0.514 | 0.694 | 0.455-0.582 | - | - | - | - |

PT: prothrombin time, APTT: activated partial thromboplastin time, Fib: fibrinogen, PC: platelet count, PCT: plateletcrit, MPV: mean platelet volume, PDW: platelet distribution width, AUC: area under receiver operating curve, CI: confidence interval, LVI: lymphovascular invasions, PNI, perineural invasions.

* indicates that the difference is statistically significant. - represents no further analysis when p > 0.05.

Supplementary Table 3. Logistic regression analysis of risk factors for LSCC

| **Items** | **β** | **SE** | **OR(95%CI)** | **p-value** |
| --- | --- | --- | --- | --- |
| **PT(S)** | 0.185 | 0.099 | 1.203(0.992-1.459) | 0.061 |
| **APTT(S)** | 0.024 | 0.021 | 1.024(0.983-1.068) | 0.256 |
| **Fib(g/L)** | 1.156 | 0.113 | 3.178(2.545-3.968) | <0.001* |
| **MPV(fL)** | -0.098 | 0.084 | 0.907(0.770-1.068 | 0.243 |

PT: prothrombin time, APTT: activated partial thromboplastin time, Fib: fibrinogen, MPV: mean platelet volume, CI: confidence interval, LSCC: laryngeal squamous cell carcinoma, S: second

* indicates that the difference is statistically significant.
